# Cerebrospinal Fluid Leak Masquerading as a Decubitus Ulcer in a Patient With Spina Bifida

**Published:** 2013-11-11

**Authors:** Erin M. Taylor, Petra M. Klinge, Stephen R. Sullivan, Helena O. Taylor

**Affiliations:** ^a^College of Physicians and Surgeons, Columbia University, New York, NY; ^b^Department of Neurosurgery, Warren Alpert Medical School of Brown University, Providence, RI; ^c^Division of Plastic and Reconstructive Surgery, Warren Alpert Medical School of Brown University, Providence, RI

**Keywords:** CSF leak, flap reconstruction, impaired wound healing, lumbosacral wound, spina bifida

## DESCRIPTION

A 38-year-old man with a history of spina bifida and T12 paraplegia presented with a nonhealing sacral wound that developed from a site adjacent to his myelomeningocele repair scar. Clear fluid drained from this pinpoint opening intermittently for years and was determined to be cerebrospinal fluid during a repeat debridement.

## QUESTIONS

1. **What factors may complicate sacral wounds in spina bifida patients?**
2. **How do you diagnose and manage a wound with a cerebrospinal fluid (CSF) leak?**
3. **What factors contribute to impaired wound healing with a cerebrospinal fluid leak?**
4. **How do you approach a non-healing sacral wound in the presence of a CSF leak?**

## DISCUSSION

Chronic, nonhealing lumbosacral wounds are a common challenge for spina bifida patients and carry significant morbidity and mortality.^1^ The most common cause of lumbosacral wounds in spina bifida patients are believed to be pressure ulcers due to a lack of sensibility and paraplegia. Pressure ulcers occur in all periods of life with a prevalence of 9.5% to 12% in myelomeningocele patients.^2^ Although pressure is believed to be the most common etiology of these wounds, we suggest that pseudomeningocele at the original myelomeningocele repair site with cerebrospinal fluid leakage should also be considered as a contributing and complicating factor in patients with a history of spinal dysraphism.

The diagnosis of a cerebrospinal fluid leak has traditionally been based on clinical observation, which includes pseudomeningocele formation, wound dehiscence, or intraoperative identification of dural tears and cerebrospinal fluid leakage.^3^ If clinically uncertain, cerebrospinal fluid leakage may be detected with immunofixation for beta-2 transferrin or the use of reagent urinary strips. Magnetic resonance imaging or cisternography with computed tomography assists in determining the location of the CSF leak and fluorescein dye may be used for localization of the lesion intraoperatively.^4^^,^^5^ Although small CSF leaks may heal with bed rest, hydration, or diverting drainage, operative repair of dural tears is the criterion standard of treatment.^6^ Direct repair typically includes the use of nonabsorbable suture with fibrin glue reinforcement. Muscle flaps, fat grafts, synthetic dural patches, and bovine pericardium tissue also have been used.^3^^,^^4^^,^^7^

Despite many clinicians' perception that CSF impedes healing, few studies have examined the effect of cerebrospinal fluid on wound healing. Cerebrospinal fluid may contribute to the softening of wound edges, as CSF is more watery than serum with only 1% of solid constituents.^8^ Cerebrospinal fluid leakage was found to effect wound healing in flap surgery in rats with histologic evidence of increased breakdown of striated muscle fibers, reactional mesenchymal tissue, calcifications, fat necrosis, ischemic coagulation necrosis, and small-diameter vessel formation.^8^ Cerebrospinal fluid fistulas occur because of a breach in the dural-arachnoid layer from incidental durotomy, trauma, or congenital abnormality. Although the majority of dural tears heal spontaneously, many factors may impair CSF fistula healing, including large dural defects, scar tissue, infection, radiation, steroid use, poor nutrition, inadequate soft tissue coverage, and elevated CSF pressures.^9^

The healing of lumbosacral wounds remains a common challenge in the treatment of patients with paraplegia. Often, the surgeon must return to complex chronic wounds multiple times for debridement, negative pressure wound therapy, and flap reconstruction. Options for surgical repair include local, regional, or free tissue transfer. Case studies on the reconstruction of sacral wounds with CSF leaks include the use of regional flaps, such as the reverse latissimus and superior gluteal artery perforator flaps, to provide thick, well-vascularized tissue coverage.^10^^,^^11^ In this case, after repair, the patient was found to have elevated intracranial pressure necessitating placement of a new ventriculoperitoneal shunt, suggesting that CSF dynamics may potentiate, or be affected by closure of these defects.

In summary, lumbosacral wounds in patients with spina bifida are more complicated than those in patients without spinal dysraphism. A CSF leak may develop at the site of myelomeningocele repair as this remains a natural and physiological weak spot. Cerebrospinal fluid may inhibit wound healing, and the dural injury puts the patient at risk for meningitis. While multidisciplinary spina bifida clinics are common for children, most adults with spina bifida must piece together care from multiple providers. This case mandated close cooperation between neurosurgery, plastic surgery, neurology, nephrology, and infectious disease. Ideally, adults with spina bifida would benefit from the same prophylactic multidisciplinary care that children receive.

## Figures and Tables

**Figure 1:**
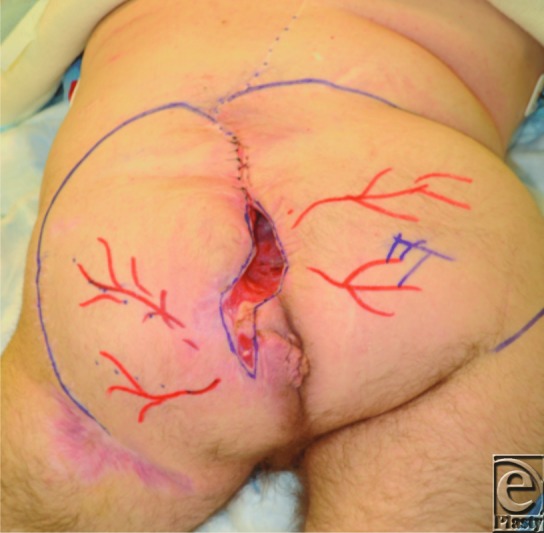
Sacral wound after debridement and prior to flap coverage, 15 × 12 cm^2^ in size that tunneled superiorly. Potential right and left rotational flaps were designed on the basis of the gluteal arteries that were identified by Doppler ultrasonography. The dotted line marks the scar from the original MMC repair used for exploration of the pseudomeningocele. MMC indicates myelomeningocele.

**Figure 2:**
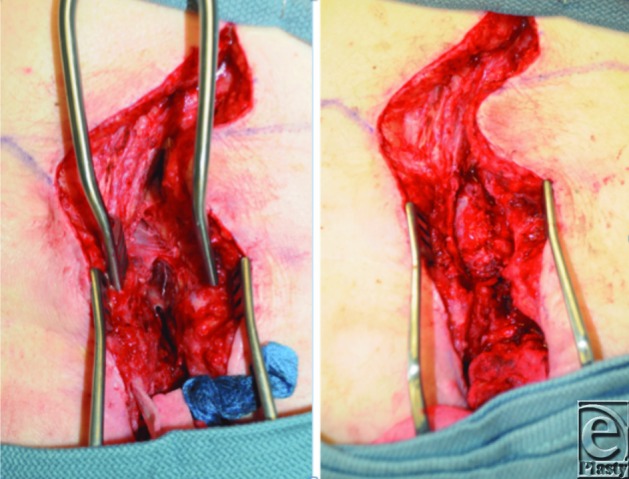
Exposure superior to the sacral wound and scar with visualization of the thecal sac and dural defect performed in conjunction with neurosurgery. (*a*) Cerebrospinal fluid leakage from the thecal MMC sac prior to repair. (*b*) Thecal sac repaired directly in a linear fashion and reinforced with fibrin glue for watertight closure. MMC indicates myelomeningocele.

**Figure 3:**
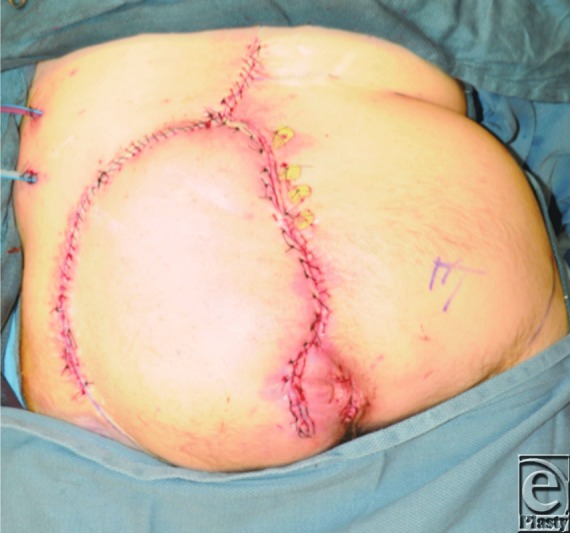
Repair of sacral wound performed with a large left gluteal fasciocutaneous rotational flap for stable and well-vascularized coverage. The medial portion of the flap was deepithelialized and secured with bolster sutures to obliterate the midline dead space.

**Figure 4:**
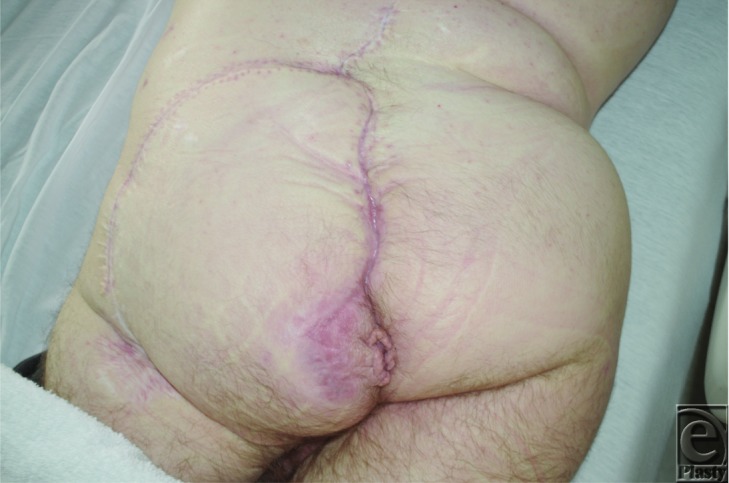
Healed wounds at 5-month follow-up, without recurrent leakage or wound breakdown.
